# Copper Coordination to the Prion Fragment (95–126): Implications for Neurodegenerative Diseases

**DOI:** 10.3390/ijms27104184

**Published:** 2026-05-08

**Authors:** Chiara Bacchella, Angelo Ferraresi, Enrico Monzani, Simone Dell’Acqua

**Affiliations:** Dipartimento di Chimica, Università di Pavia, Via Taramelli 12, 27100 Pavia, Italy; angelo.ferraresi01@universitadipavia.it (A.F.); enrico.monzani@unipv.it (E.M.)

**Keywords:** prion peptides, copper, dopamine, oxidative stress, protein-membrane interaction

## Abstract

The causative event in transmissible spongiform encephalopathies is the misfolding of the prion protein (PrP), a process influenced, in a way that is not yet fully understood, by transition metal ions, particularly copper, which modulate folding, aggregation, and redox activity. In this study, we investigated the interaction of copper(II) ions with the prion fragment PrP(95–126), which includes the non-octarepeat high-affinity sites His96 and His111, as well as an amyloidogenic tail involved in PrP misfolding and membrane interaction. UV–vis and circular dichroism analyses revealed the predominant formation of a 1:1 Cu/PrP(95–126) complex, accompanied by modest restructuring, consistent with an increased aggregation propensity upon copper binding. The Cu/PrP(95–126) complexes exhibited limited redox activity toward catechol substrates, which was further reduced in membrane-mimetic systems such as SDS micelles and large unilamellar vesicles (LUVs). His96 appears not to play a critical role in copper coordination or redox activation. This study explores the coordination modes and reactivity of copper(II) with PrP, as well as employing a membrane mimic, aspects that are still highly controversial in the literature, providing insights for further in vitro studies.

## 1. Introduction

The cellular prion protein (PrP^C^) is a highly conserved glycosylphosphatidylinositol (GPI)-anchored glycoprotein that is widely expressed in mammals, with particularly high levels in the central nervous system [1]. Under physiological conditions, PrP^C^ is predominantly α-helical and localized on the surface of cells; however, it can undergo a conformational transition into a misfolded, β-sheet-rich isoform known as the “scrapie” form (PrP^Sc^) [2], which represents the infectious agent responsible for transmissible spongiform encephalopathies (TSEs), including Creutzfeldt–Jakob disease in humans [3,4,5]. These fatal neurodegenerative disorders are characterized by spongiform degeneration of the brain, amyloid deposition, neuronal loss, and synaptic dysfunction [6].

In pathological conditions, the conversion of PrP^C^ into PrP^Sc^ involves a structural rearrangement from an α-helical to a β-sheet-enriched conformation. This altered folding renders the protein prone to aggregation, partially resistant to proteolytic degradation, capable of self-propagation, and ultimately neurotoxic [7].

**Figure 1:**
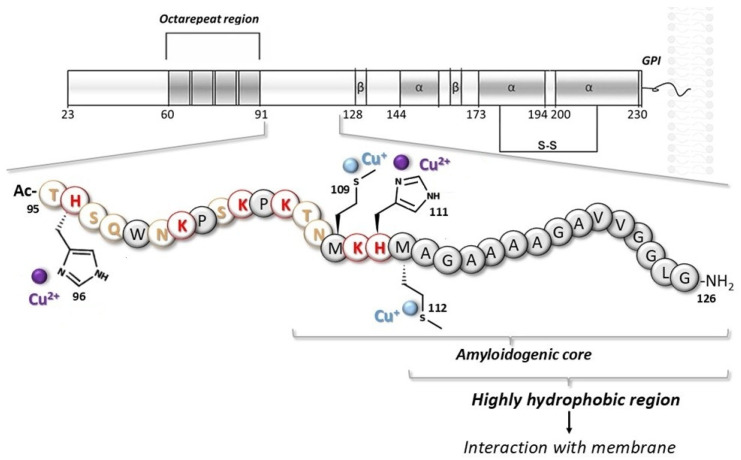
Schematic representation of the main functional domains of the prion protein, with an expansion of the sequence used in this study. Side chains of residues involved in copper interaction are also shown.

Structurally, PrP^C^ consists of a flexible, intrinsically disordered N-terminal domain and a globular C-terminal domain stabilized by a single disulfide bond (Figure 1). Despite its central involvement in prion diseases, the normal physiological function of PrP^C^ remains incompletely understood. Notably, PrP knockout mice display minimal abnormalities under normal conditions, suggesting that PrP^C^ may be involved in complex regulatory pathways, potentially including compensatory mechanisms that mask its functional loss [8].

One of the most extensively studied proposed functions of PrP^C^ involves its ability to bind transition metal ions. The N-terminal region contains an octarepeat (OR) domain composed of four tandem repeats of the sequence PHGGGWGQ. Each repeat includes a histidine residue, which plays a crucial role in coordinating metal ions. This highly conserved domain binds copper, zinc, and manganese ions, with particularly high affinity for copper ions in both its redox states [9,10,11,12]. In addition to the octarepeat region, a second high-affinity copper-binding site is located outside the repeats and involves His96 and His111, together with Met110 and Met112, residues that are particularly important for stabilizing copper(I) [13].

Overall, PrP^C^ can bind up to six Cu^2+^ ions, influencing its endocytosis and cell-surface expression. Hijazi and colleagues have observed that when scrapie-infected neuroblastoma cells were cultured in the presence of copper, the accumulation of PrP^Sc^ in these cells was markedly reduced, and a significant delay in the onset of the disease was observed. The authors suggest that copper exerts a protective effect from prion infection, probably because of the rearrangement and regulation of PrP^C^ location [14].

Moreover, Cu binding finely modulates the prion conformation, stability and fibril structure: for instance, when one molar equivalent of Cu^2+^ is bound, PrP fibrils adopt a core structure distinct from that formed in the absence of copper [15]. This conformational change appears to be primarily driven by copper coordination (particularly to His96 and His111, together with His140), which plays a key role in defining the altered fibril architecture [16]. Copper was indeed shown to promote the conversion of PrP^C^ into a partially protease-resistant peptide [17,18] and to accelerate the aggregation of the neurotoxic peptide PrP(106–126), the peptide comprising the 106–126 sequence of the protein [19].

Evidence supports roles for the prion protein in copper homeostasis, antioxidant defense, and copper-dependent neuroprotection, including modulation of NMDA receptor activity [20]. Within this context, recent work by Petruzzelli et al. provided compelling evidence that PrP^C^ can also exacerbate copper toxicity in Wilson disease, a genetic disorder caused by loss of the copper transporter ATP7B. The authors demonstrated, both in vitro and in vivo, that ATP7B deficiency leads to upregulation of PrP, which enhances copper uptake and intracellular accumulation, thereby promoting oxidative stress and cellular injury. Importantly, reducing PrP expression significantly attenuated copper-induced toxicity. These findings position PrP not merely as a passive copper-binding protein but as an active regulator of copper metabolism, beyond its established role in prion diseases [21].

Previous studies performed on copper(II) complexes of prion peptides containing 3, 4, and 6 histidine residues in the sequences of PrP(84–114), PrP(76–114), and PrP(60–114), respectively, revealed a particular order of affinity of the various His sites for the metal complexation His111 > His96 >> His(octarepeat) [22,23,24]. Even though the individual affinity constant of a single OR site is low, the cooperative effect of multiple His residues increases effective binding at low copper concentrations because the macrochelate stabilizes the complex. On the other hand, as local Cu^2+^ concentrations rise (such as in pathological states), the OR sites become quickly saturated, and copper binds to other histidine residues outside the OR domain, which, although they do not have a macrochelate effect, are characterized by a higher binding affinity for Cu^2+^ [25].

For this reason, we have chosen to explore the nature of copper interaction with a prion fragment that includes the region outside the octarepeat, specifically residues 95–126 (Figure 1).

In addition to its ability to bind copper ions, the 95–126 sequence is considered critical in early conformational transitions, leading to misfolding and structural changes in this region, which influence β-sheet formation and aggregation. Indeed, this sequence contains a hydrophobic segment known as the amyloidogenic sequence, which is highly prone to fibrillation. This peptide portion has also been indicated as a possible site of interaction with the amyloid-β peptide, particularly in the 95–110 fragment [26,27]. Studies conducted with the shorter fragment, PrP(106–126), revealed a high content of β-structures [28], which were responsible for its increased resistance to enzymatic degradation and its high susceptibility to fibrillation [29].

The pathological structural conversion is further promoted by the high susceptibility of this region to oxidative reactions, due to the presence of two methionine residues. The sulfur atom in the methionine side chain is particularly prone to oxidation, leading to the formation of methionine sulfoxide and, under stronger oxidative conditions, methionine sulfone. These oxidative modifications alter the local hydrophobicity and disrupt intramolecular interactions, thereby destabilizing the native conformation of the prion protein and facilitating its aggregation [30].

In addition, the 95–126 region plays a crucial role in membrane interaction: it has been observed to mediate neuronal apoptosis, likely by altering membrane permeability through the formation of nonselective channels [31,32,33].

Although the prion protein was discovered in 1982 (which later earned Stanley B. Prusiner the Nobel Prize in 1997) and its interaction with copper has been recognized since the late 1990s, contrasting studies continue to be published regarding the functional implications of this interaction and the precise mechanisms by which it contributes to prion pathology. For instance, Torres et al. reported that the substitution of His95 (corresponding to human His96) with tyrosine in mice induces neurodegeneration [34]. In contrast, Giachin et al. demonstrated in vitro that the same mutation stabilizes the cellular form of the prion protein, reducing its propensity for misfolding and pathological deposition by altering copper coordination [35].

These apparently opposing findings highlight the complexity of copper–PrP interactions and provide a strong rationale for the present study.

In this work, we aim to determine the role of this confined region in copper sequestration and metal redox activation in the presence of external reducing molecules, such as catechols. Additionally, we seek to gather preliminary data on the catalytic behavior of this metal complex when membrane models, like sodium dodecyl sulfate (SDS) micelles or large unilamellar vesicles (LUVs), are present in solution and interact with the peptide.

## 2. Results and Discussion

### 2.1. Interaction Between Copper and PrP(95–126)

**Figure 2:**
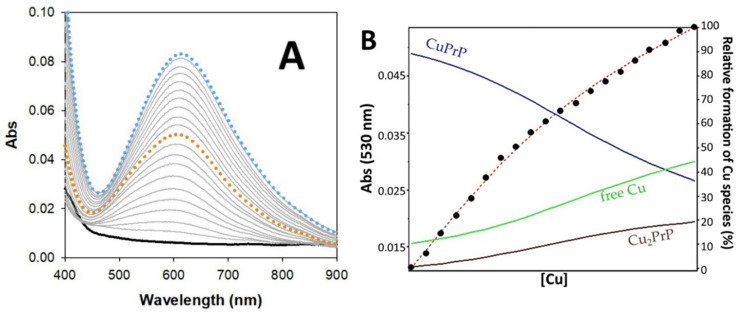
(**A**) Titration of PrP(95–126) (0.5 mM) in 50 mM HEPES buffer at pH 7.4 and 25 °C vs. copper(II) (0–1 mM, by successive additions of 0.05 mM Cu^2+^); dotted orange and light blue spectra represent Cu vs. PrP 1:1 and 2:1, respectively. Panel (**B**) shows the titration points at 530 nm superimposed on the species distribution diagram. Solid curves show the relative percentages of the following species: Cu alone, 1:1 and 2:1 Cu/PrP(95–126) species; the *x*-axis linearly represents the concentration range used (0–1 mM).

To characterize the formation of the complex Cu/PrP(95–126) at physiological pH, we followed the UV–vis spectroscopy titration of the peptide vs. increasing equivalents of copper(II) ions in 50 mM HEPES buffer solution at pH 7.4 (Figure 2). The data suggest that the binding is dominated by formation of the species 1:1 Cu/PrP(95–126), with an absorption maximum at 608 nm, and the formation equilibrium is governed by a modest binding constant (logK = 4.26 ± 0.02—Table 1). By enhancing the concentration of copper(II) in solution, we observe that the equilibrium is moved towards the species 2:1 Cu/PrP(95–126), leading to a modest red shift of the absorption band to 618 nm. This second equilibrium is characterized by a low binding constant (logK = 2.78 ± 0.03), indicating that multinuclear complex formation is possible, but only under conditions of excess copper. However, considering that copper concentrations can reach approximately 0.250 mM during neuronal depolarization, this type of interaction may become biologically relevant [36]. On the other hand, when the titration is performed in reverse order, using an excess of the prion fragment, no formation of a Cu/(PrP(95–126))_2_ system is observed (Figure S1). This finding excludes the formation of complexes in which copper is positioned as a bridging ion between two peptide molecules, although copper may indirectly mediate intermolecular interactions, as described below.

A modest value of the constant associated with the formation of the mononuclear complex (Table 1) is also observed with a shorter peptide, PrP(91–114), which lacks the hydrophobic tail (not directly involved in copper coordination). The longer peptides PrP(76–114) and PrP(84–114), which contain one and two octarepeats, respectively, exhibit higher binding affinity constants, suggesting that, at physiological pH, copper may be simultaneously bound to various histidine residues both within and outside the octarepeat sequence [22,23]. Indeed, Cu binding to non-octarepeat His residues may have a cooperative effect for copper coordination to the octarepeat region [37].

Interestingly, the LogK values for copper binding affinities of PrP(95–126) and PrP(106–114) (which lacks the His96 residue), are not significantly different, suggesting that His96 may not play a relevant role in binding Cu(II) ions. Indeed, there are discrepancies in the literature regarding the potential involvement of this residue in the so-called fifth Cu(II)-binding site. For instance, an EPR study proposed that His96 is uniquely involved in Cu(II) coordination [38], while an EXAFS study suggested that both His96 and His111, along with Met residues, participate simultaneously [39]. On the other hand, other analyses exclude the possibility of a macrochelate involving both His96 and His111 due to the steric hindrance provided by the two Pro residues at positions 102 and 105 [40]. Quintanar et al. studied the interaction of copper(II) with the PrP(92–115) peptide and proposed that His111 exhibits a higher affinity for Cu^2+^, with additional contributions of the deprotonated backbone amide nitrogens and a carbonyl oxygen (Met109), leading to an equilibrium between the 3N1O and 4N coordination modes [41].

**Table 1:** Experimental and published LogK values of Cu^2+^ complexes with PrP(95–126) and other PrP fragments shown in the literature.

	logK	Ref.
PrP(95–126) + Cu^2+^ ⇄ [Cu-PrP(95–126)]	4.26	
PrP(76–114) + Cu^2+^ ⇄ [Cu-PrP(76–114)]	10.07	[23]
PrP(84–114) + Cu^2+^ ⇄ [Cu-PrP(84–114)]	6.48	[22]
PrP(91–115) + Cu^2+^ ⇄ [Cu-PrP(91–114)]	4.84	[42]
PrP(106–114) + Cu^2+^ ⇄ [Cu-PrP(106–114)]	4.06	[43]

Copper interaction with the PrP(95–126) peptide was also evaluated in terms of structural changes following metal binding. We therefore performed conformational studies of PrP(95–126) using far-UV circular dichroism analysis. The results showed that the peptide has a largely random coil structure when free in solution (Figure S2). However, upon addition of increasing amounts of copper(II) ions, its structure undergoes modest rearrangement toward a more rigid conformation with a slight increase in β-sheet content. This finding is consistent with the observed increase in aggregation propensity promoted by copper interaction, as demonstrated by turbidimetry studies conducted over a 7-day period (Figure S3).

The sequence spanning residues 106–114 has been proposed as the region with the highest affinity for copper ion (in both redox states) binding in PrP [43]; this suggests an important involvement of this domain in metal redox cycling under physiological conditions. However, our previous kinetic analyses indicates that the coordination of PrP(106–114) has a minimal impact on Cu oxidative reactivity [44]. Therefore, if His96 is not essential for copper binding, we would expect the Cu/PrP(95–126) system to exhibit similar neurotoxicity to that observed for Cu/PrP(106–114) in terms of catalyzed oxidative damage to external substrates (such as catechol molecules).

### 2.2. Reactivity of Cu/PrP(95–126) Complex and Its Behavior in Membrane-like Systems

As previously done for other Cu-peptide complexes [44,45,46,47,48,49,50,51,52,53,54,55,56,57,58,59], we assessed the redox activity of the metallospecies under physiological conditions. The oxidation of reducing agents (i.e., catechols) was spectrophotometrically studied at room temperature by exposing the solutions to air oxygen. The formation of oxidative products (which can also provide information about reactive oxygen species (ROS) production) is governed by the efficiency of Cu redox cycling. This catalytic efficiency can be influenced by the presence of coordinating ligands, including the peptide itself. 4-methylcatechol (MC) was chosen as a suitable model for biologically relevant catecholamines (such as dopamine, norepinephrine, and epinephrine), as it gives rise to a simpler mixture of oxidation products [47,60].

**Figure 3:**
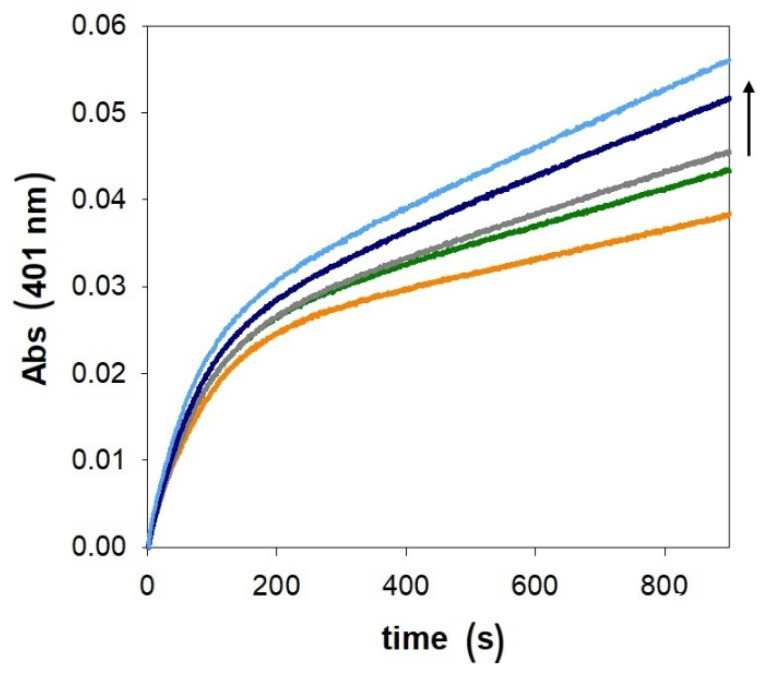
Kinetic profiles of MC (3 mM) oxidation with time in 50 mM HEPES buffer at pH 7.4 and 25 °C promoted by copper(II) alone (25 µM, orange trace) in the presence of PrP(95–126) (25 µM, green; 50 µM, grey; 75 µM, blue and 100 µM, light blue).

As shown in Figure 3, the copper(II)-mediated oxidation of MC to 4-methylquinone (MQ) displays a biphasic kinetic pattern that is only moderately affected by increasing concentrations of PrP(95–126). In comparison to previously published data from our group on other prion fragments (PrP(76–144), PrP(84–114), and PrP(106–114) [44], which contain 4, 3, and 1 His residues, respectively), it is evident that the reactivity of Cu-PrP(95–126) follows a similar catalytic trend to that observed with the shorter, 106–114, peptide. As shown by the modest Cu binding affinity and by the low Cu/PrP oxidative efficiency, it may be reasonable to conclude that the role of His96 in metal coordination and in the activation of the Cu redox cycle is minimal. Additionally, we performed the kinetic study in the presence of PrP(95–114) (Figure S4), a shorter and more hydrophilic peptide, with respect to PrP(95–126), to confirm that the reduced Cu activation is not due to the high steric hindrance from the long hydrophobic tail (113–126) of PrP(95–126), which may partially impede the Cu/PrP interaction.

It is important to note that all oxidation experiments were carried out in the presence of a large excess of the catechol substrate (at millimolar concentration vs. micromolar values of the catalyst and PrP), which is a potential ligand for copper(II) ions. On the other hand, by reducing [MC] by one order of magnitude to decrease the competition for Cu binding between PrP and the substrate (Figure S5), the effect of the peptide remains marginal, primarily limited to the second phase of the catalysis. Indeed, the biphasic kinetic trace can be razionalized by applying the reaction mechanism previously proposed for the oxidation of catechols by Cu-peptide complexes [50] to the Cu-PrP(95–126) system.

In the initial phase of the reaction, MQ is formed and it accumulates in solution, but after about 100 s, it is consumed by the formation of further addition products. These species absorb at higher, with respect to the reading value, wavelengths, giving rise to a reduction in the slope of the kinetic traces shown in Figure 3. A further effect ruling the biphasic behavior is that the initial reaction between copper(II) and MC is fast, whereas the following reaction between the formed copper(I) complex and O_2_ is slow. The latter reaction is the rate-limiting step of the catalytic cycle since it requires the formation of the key ternary complex [Cu-PrP-catechol-O_2_], essential for completing the two-electron transfer. The efficiency of copper(I) oxygenation is tightly associated with the geometry and coordination sphere of the metal ion. The presence in PrP(95–126) of His111 and two Met residues provides a suitable copper(I) binding site (it prefers low coordination numbers and soft ligands). As a consequence, the limited effect of PrP(95–126) on the second-phase reaction rates is not surprising.

The low efficiency in MC oxidation of Cu/PrP(95–126) was also verified by estimating the catalytic constants K_M_ and *k_cat_*: the study was performed by varying the concentration of the substrate (0–4 mM) in the presence of fixed amounts of catalyst (copper alone or Cu/PrP(95–126) complex, 1:1, 25 µM) (Figure S6). Fitting the initial reaction rates of MC oxidation (Figure S7) catalyzed by the Cu/PrP(95–126) system through the Michaelis–Menten equation yields the following values: K_M_ (mM) = 0.33 ± 0.03 and *k_cat_* (s^−1^) = (3.72 ± 0.04) × 10^−3^.

By comparing these values with those obtained in the absence of PrP peptides (Table 2), it becomes clear that although prion coordination modestly enhances the *k*_cat_ values, it also leads to an increase in the K_M_ value compared to “free” copper(II). This suggests that PrP coordination negatively regulates the formation of the Cu/MC system and leads to an almost unchanged catalytic efficiency of copper, as shown by the similar *k*_cat_/K_M_ ratio (Table 2). This result provides evidence for the ineffective role of His96 in copper coordination and participation in its redox cycle, as well as for the negative influence of the hydrophobic tail, which likely hinders interaction with the substrate. Interestingly, the catalytic efficiency of Cu/PrP(95–126) is lower than that of Cu/PrP(106–114) (which contains only one histidine), suggesting that steric hindrance predominates over potential differences in copper coordination.

The presence of the amyloidogenic tail not only affects the accessibility of the bound copper ion, but may also mediate the interactions of the Cu-complex with cellular lipids. Given the importance of PrP–membrane interactions [61,62], it is crucial to understand how the presence of phospholipids may influence the binding mode and redox reactivity of Cu/PrP.

**Figure 4:**
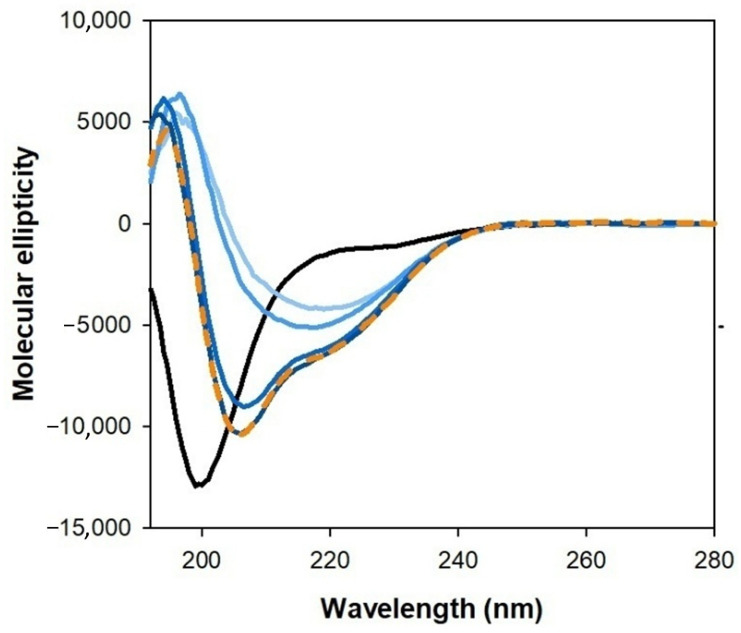
CD spectra in the far-UV region of PrP(95–126) alone (10 µM, black spectrum), in 5 mM phosphate-buffered solution at pH 7.4, with the addition of SDS (2 mM, 5 mM, 10 and 20 mM—scale of blue) and copper(II) (9.5 µM, dashed orange).

We therefore first characterized the structural changes occurring when the copper complex interacts with simple membrane mimic systems, such as sodium dodecyl sulfate (SDS) micelles. The significant effect of the PrP(95–126)/SDS interaction, likely involving the amyloidogenic domain of the peptide (particularly the hydrophobic sequence 113–126), is evident in the CD spectra of Figure 4, which show structural changes in the peptide backbone upon addition of SDS.

As described before, in aqueous solution, PrP(95–126) adopts a random conformation, and copper(II) binding induces only small structural changes, indicated by an appreciable reduction in the intensity of the CD signal, without any significant shift (Figure S8). On the other hand, the addition of SDS induces conformational changes, resulting in the appearance of two dichroic signals at 208 and 222 nm, typical of an increase in α-helix conformation. This effect is similar to that observed with amyloid-β peptides [46] and α-synuclein protein [51] in SDS, both of which contain highly hydrophobic domains capable of interacting with micelles and other membrane-like systems. In these cases, membrane association causes significant conformational changes that limit the mobility of the amino acid backbone, thereby limiting interactions with metal ions. This behavior was shown to strongly influence the redox activity of the complex in membrane environments, partially confining and sequestering the C-terminal copper(I) binding site within or at the surface of the micelles [46].

Indeed, our previous study showed that the redox behavior of Cu/PrP peptide complexes is highly dependent on the stabilization of the Cu(I) intermediate, particularly when the reduced metal ion is anchored at the MetXxxYyyHisMet site and confined within micelles. Under these conditions, the reactivity of the reduced intermediate (and its interaction with dioxygen) is significantly decreased [46].

**Figure 5:**
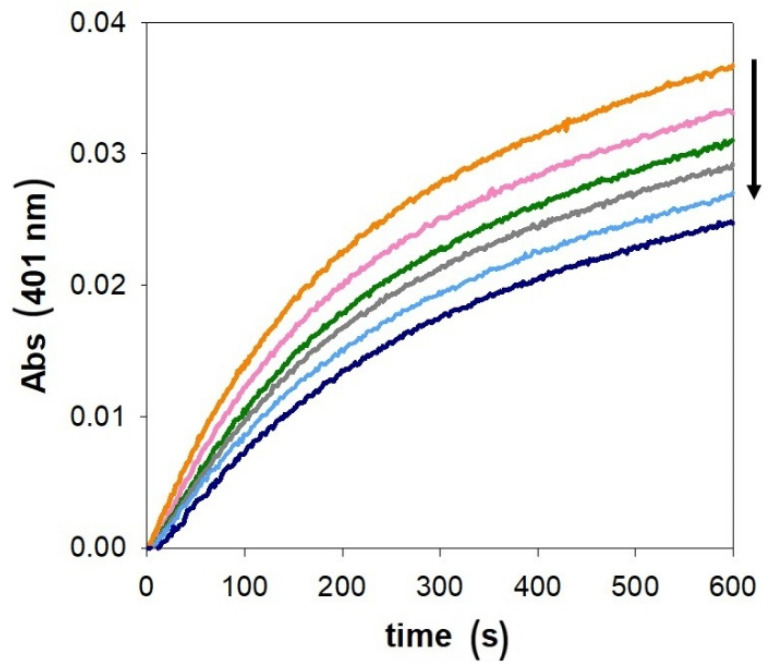
Absorbance changes with time in the MC (3 mM) oxidation in micellar solution of SDS (20 mM) in 50 mM HEPES buffer at pH 7.4 and 25 °C promoted by copper(II) alone (25 µM, orange trace) in the presence of PrP(95–126) (12.5 µM, pink; 25 µM, green; 50 µM, grey; 75 µM, light blue, 100 µM, blue).

For these reasons, the catalytic activity of Cu/PrP(95–126) in the oxidation of MC, an indicator of the redox properties of the complex, was investigated not only in aqueous solutions but also in micellar systems. SDS above its critical micelle concentration was used to simulate a membrane-like environment [63]. Unlike the data obtained without SDS (see Figure 3), increasing the amount of coordinating prion fragment resulted in a decrease in the catalytic reaction rate (Figure 5), likely due to the partial trapping of the metal complex within the micelles.

As done in previous works [46,50], we also assayed the reactivity of the copper complex by monitoring oxidative modifications of the peptide over time using HPLC/MS analysis. Indeed, these oxidative reactions can competitively target either an external substrate or susceptible amino acid side chains. When oxidative modifications of the peptide were assayed in SDS and compared with those obtained in aqueous medium, higher susceptibility and faster tendency toward oxidation were observed in micelles (Figure S9). In particular, a higher percentage of oxidations at His111 and Met109 was detected in copper–peptide complexes confined within micelles (Table S1). This result suggests that: (i) the membrane controls the accessibility to the metal ion and the interaction between the copper complex and the substrate, likely due to the different localization of the copper–peptide complex and the neutral (with in MC) or charged (as in dopamine, see below) catechol moiety within the micellar environment, and (ii) membrane interaction likely confines copper near His111 and Met109, thereby increasing the susceptibility of these residues to oxidation. Because of these effects, the overall reactivity toward the substrate appears reduced. However, the redox-cycling metal, mainly trapped at the His111 site, remains sufficiently reactive to oxidize nearby amino acids likely via a ROS-dependent mechanism, as described by Cheignon et al. for amyloid sequences [64].

When the catalytic constants were measured in SDS micelles, a trend opposite to that observed in aqueous solution was observed. As shown in Figures S10 and S11, where the MC concentration is increased while the catalyst concentration remains constant (copper(II) or the Cu/PrP(95–126) complex at 25 µM), the presence of the coordinating peptide, which is partially trapped in the membrane, reduces the reaction rates. While the free copper(II)-MC system shows similar catalytic efficiency with or without SDS (as shown by the similar *k_cat_*/K_M_ ratio value), since the substrate does not significantly interact with the micelle surface or core, the reaction catalyzed by Cu/PrP(95–126) is partially quenched by SDS compared to what was observed in aqueous solution. Specifically, when Cu/PrP(95–126) is in the micelles, its interaction with both the substrate and dioxygen is partially hindered (Table 2).

To study a more realistic (with respect to SDS micelle) membrane-mimetic system, we employed Large Unilamellar Vesicles (LUVs) formed by mixing a neutral (2-oleoyl-1-palmitoyl-sn-glycero-3-phosphocholine—POPC) and a negatively charged (1-palmitoyl-2-oleoyl-sn-glycero-3-phospho-L-serine—POPS) phospholipid in a 9:1 ratio [65,66].

**Figure 6:**
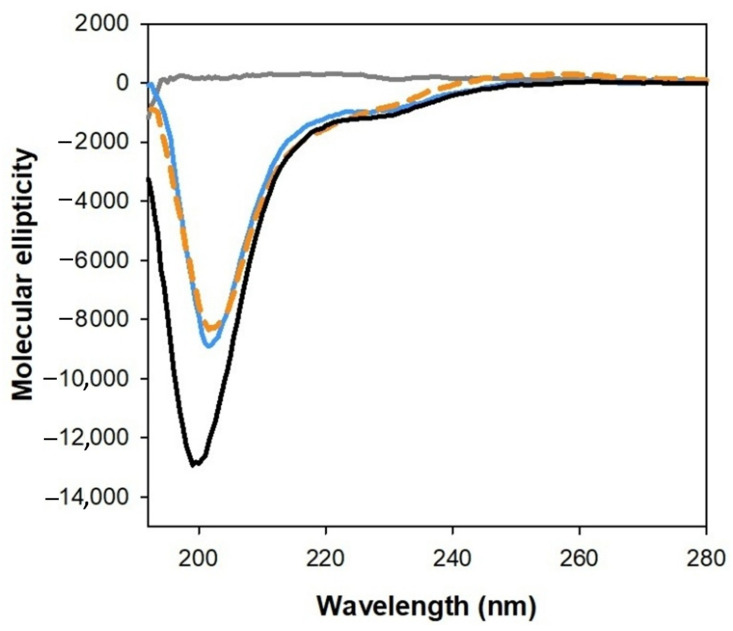
CD spectra in the far-UV region of PrP(95–126) alone (10 µM, black spectrum), in 5 mM phosphate-buffered solution at pH 7.4, with the addition of LUVs (100 µM, light blue) and copper(II) (9.5 µM, dashed orange). LUVs in buffer are shown as grey spectrum.

As performed previously, we first investigated the structural influence of LUVs on the peptide, both alone and bound to copper ions, using CD spectroscopy. Compared with what was observed with negatively charged SDS micelles, the peptide interacting with LUVs undergoes less pronounced conformational changes (Figure 6). It should be noted that the concentrations of the two membrane models differ by two orders of magnitude, and their surface properties vary significantly in terms of charge, size (SUVs are typically 15–30 nm in diameter, whereas LUVs range from 100–200 nm or larger), and membrane curvature, which is lower in LUVs [67].

The choice of phospholipids used to prepare LUVs and their ratio strongly influences the interaction affinity with PrP peptides [68,69,70]; in our case, the use of an almost neutral membrane surface, containing only ~10% negatively charged lipids, likely results in weak electrostatic attraction toward the hydrophilic flexible domain of the PrP(95–126) peptide. When the HPLC/MS analysis previously performed in buffer and SDS micelles was repeated in the presence of LUVs, it was not possible to separate the precipitated phospholipids from the peptide: this observation may suggest that the prion peptide still interacts, albeit modestly, with this membrane system (Figure S9C).

We investigated the MC oxidation promoted by free and PrP(95–126)-bound Cu^2+^ ions, both in the presence and absence of LUVs, and we found that the presence of the coordinating peptide trapped in the vesicles has a slightly modest effect on copper(II) reactivity, mainly in the first seconds of catalysis, resulting in a transient initial slowdown of the reaction (Figure S12). LUVs appear to strongly interact with the uncharged substrate, likely trapping MC within the vesicle surface (which is predominantly uncharged) or in its inner core, making it less accessible and reactive to copper. This effect consequently leads to a quenching of overall reactivity. LUVs might also alter binding affinities of copper(II) for PrP(95–126) and for MC in complex ways, but these may contribute to the quenching of the reaction rates. Although HPLC/MS data could not be obtained, we can hypothesize that a mechanism similar to that observed in SDS may occur, in which oxidation of amino acids modifies the properties of the copper-binding site: such modifications may affect copper stabilization and reactivity, potentially leading to a restart of catalysis following ligand oxidation (as detected in Figure S12).

Similar results were observed starting from the reduced Cu(I)-complex: tetrakis(acetonitrile)copper(I) hexafluorophosphate (25 µM) was first dissolved in pure acetonitrile and, after five vacuum/argon cycles, added to the reaction mixture containing the substrate and the peptide (25 µM). In this case, only a small stabilization of Cu(I) by PrP coordination was observed in both the aqueous and lipid phases (Figure S13—blue and dotted orange traces, respectively). The slight delay of substrate oxidation may be attributed to a modest stabilization of the copper(I) ion bound to the Met site of the peptide, favored by the LUV medium, which likely hampers efficient oxygen binding.

The relationship between the kinetic trend and the chemical properties of the substrate was investigated by replacing the neutral 4-methylcatechol with the positively charged catecholamine dopamine, which is naturally present in neurons as a neurotransmitter. We therefore investigated the oxidation of dopamine, which leads to the formation of dopaminochrome, spectrophotometrically monitored by following the increase in absorbance at 475 nm.

**Figure 7:**
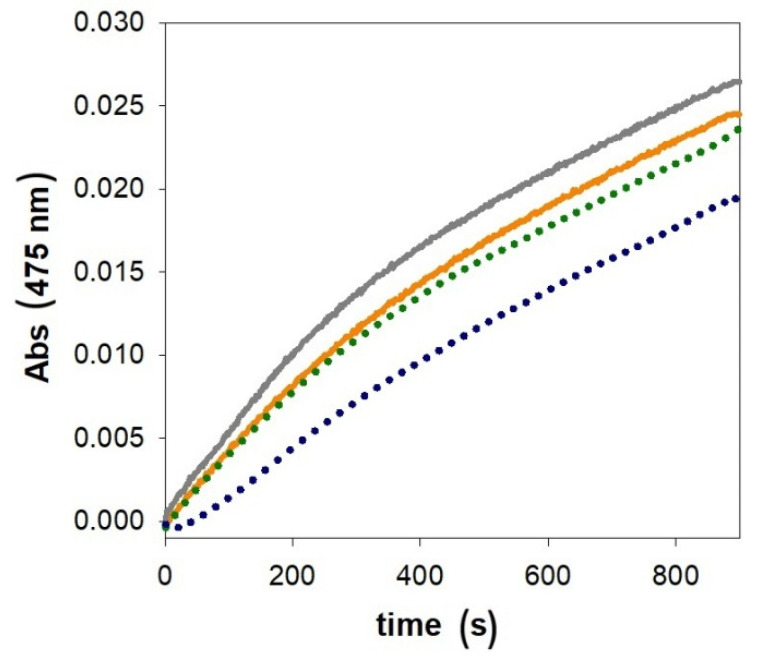
Absorbance changes with time in dopamine (3 mM) oxidation in 50 mM HEPES buffer at pH 7.4 and 25 °C promoted by copper(II) alone (25 µM) without (orange trace)/with LUVs (1.5 mM, dotted green) and in the presence of PrP(95–126) (25 µM) without (grey)/with LUVs (1.5 mM, dotted blue).

Although the prion fragment (95–126) promotes the redox cycling of copper with dopamine in aqueous solution (Figure S14) only to a limited extent, the reactivity of the metal complex in LUVs is slightly reduced, suggesting a modest stabilization/inactivation of the catalytically competent species inside or on the surface of the vesicles (Figure 7).

Differently from the results previously obtained by our group in SDS micelles (where the completely negatively charged surface influenced the distribution and reactivity of the substrate) [46,51], LUVs do not interact with dopamine, and this is likely due to the low content of negatively charged phospholipid heads. As a consequence, the vesicles do not substantially affect the availability of dopamine in solution. Therefore, the observed changes in reactivity cannot be attributed to sequestration of dopamine by the vesicles, but rather to differences in the reactivity of the Cu/PrP(95–126) complex in the presence of LUVs (Figure 7). This effect remains relatively modest, likely because of the limited interaction between the Cu/PrP(95–126) complex and the vesicles, as well as the competition for copper binding between the peptide (which displays a moderate binding constant, as previously shown) and the catecholamine. As a result, a fraction of the Cu/DA complex could remain reactive in solution, outside the membrane environment.

## 3. Materials and Methods

**Peptide Synthesis and Purification**. The synthesis of PrP(95–126) (Ac-_95_THSQWNKPSKPKTNMKHMAGAAAAGAVVGGLG_126_-NH_2_; MW 3245 a.m.u.) was performed by using the standard fluorenyl methoxycarbonyl (Fmoc) solid-phase synthesis in dimethylformamide (DMF) [71]. Low-loading Rink-amide resin MBHA (substitution 0.36 mmol/g—Sigma-Aldrich, St. Louis, MO, USA), which yielded the peptide amidated at the *C*-terminus, was used as the polymeric support. The deprotection of the resin and the Fmoc group from each amino acid was performed with 20 mL of 20% (*v*/*v*) piperidine in DMF, repeating the reaction twice, for 3 and 7 min. Each amino acid (2 mol equiv. vs. resin sites) was added in the presence of 2 equiv. of N-hydroxybenzotriazole, 2 equiv. of benzotriazol-1-yloxytripyrrolidinophosphonium hexafluorophosphate, and ∼2 equiv. of N,N-diisopropyl ethylamine. The coupling reaction was carried for at least 45 min in a mixture of DMF:NMP (5:2). After recoupling of each amino acid, a capping step was performed by using 20 mL of 4.7% acetic anhydride and 4% of pyridine in DMF; the resin was washed with DMF, dichloromethane, and isopropanol. At the end of the synthesis, the protective groups of the side chains of the amino acids were removed with a solution of 92.5% trifluoroacetic acid (TFA, 15 mL), triisopropyl silane (2.5%), water (2.5%) and 1,2-ethanedithiol (2.5%). After stirring for 3 h, cold diethyl ether was added to precipitate the peptide, and the mixture was filtered; then, it was dissolved in water with 0.1% TFA and purified by HPLC, using a 0−70% linear gradient of 0.1% TFA in water to 0.1% TFA in CH_3_CN over 40 min (flow rate of 2.5 mL/min, loop 1 mL) as the eluent. The identity of the peptide was confirmed by ESI-MS (Thermo-Finnigan, San Jose, CA, USA). ESI-MS data (direct injection, MeOH, positive ion mode, capillary temperature 200 °C): *m*/*z* 1082.5 (PrPH_3_)^3+^, 812.5 (PrPH_4_)^4+^, 650 (PrPH_5_)^5+^.

**Preparation of LUVs.** A 9:1 mixture of PC:PS (total lipid concentration = 2.4 × 10^−6^ mol) was dissolved in 1.5 mL HEPES buffer (50 mM, pH 7.4). The mixture was vortexed for 5 min, resulting in a milky solution. Next, five rapid cycles of N_2_-freezing and heating in a 50 °C water bath were performed, with sonication for a few minutes after each cycle to achieve a clear solution. The vesicle population was then passed through 200 nm filters. LUV solution was freshly prepared daily, while the stock solution was dissolved in chloroform, evaporated, dried overnight, and stored under argon at −20 °C; the daily solution was assayed to determine its actual concentration before preparation of vesicles. The stock solution concentration, determined as total phosphorus by UV–Vis spectroscopy (as described in the “HPLC-ESI/MS studies” section), was measured according to the method reported by Chen et al. [72,73].

**Binding studies.** Spectrophotometric titration was performed (i) in the presence of fixed amount of PrP(95–126) (0.5 mM) vs. increasing amount of copper(II) nitrate trihydrate (from 0 to 2 equiv.—1 mM), or (ii) in the presence of fixed amount of copper(II) (0.25 mM) vs. increasing amount of PrP(95–126) (from 0 to 2 equiv.—1 mM), in 50 mM HEPES buffer at pH 7.4. All experiments were performed in thermostated cells at 25 °C. Titration data were processed with HypSpec Software (2008). The peptide solution was quantified via UV–vis (ε_280_ = 5500 M^−1^cm^−1^), while the concentration of copper stock solution was estimated by titration with EDTA.

**Turbidimetry assay.** Aggregation was assessed through turbidity measurements. PBS buffer at pH 7.4 (137 mM NaCl, 3 mM KCl, 10 mM Na_2_HPO_4_, and 2 mM KH_2_PO_4_, ionic strength ∼160 mM) was treated with Chelex resin under stirring for 24 h. The resin was then removed by filtering the solution through a 0.2 μm filter. The samples, containing PrP(95–126) (0.3 mM) and copper(II) (0–0.3 mM), were incubated at 37 °C under stirring in the dark for seven days. Aggregation was monitored by measuring the turbidity of the solutions at 400 nm at different time points (0, 1, 2, 3, 4, 5 and 7 days) using a UV–vis spectrophotometer (Agilent Technologies, Santa Clara, CA, USA).

**Kinetic studies.** The catechol oxidation catalyzed by copper(II) complexes was investigated at 20 °C in a 50 mM HEPES buffer at pH 7.4, saturated with atmospheric oxygen. The reaction was monitored via UV–visible spectroscopy, following the 4-methylquinone (MQ) band at 401 nm (ε = 1550 M^−1^ cm^−1^) for 4-methylcatechol (MC) oxidation or the dopaminochrome band at 475 nm (ε = 3300 M^−1^ cm^−1^) for dopamine (DA) oxidation. Substrate concentrations were maintained at 0.3 or 3 mM, while the catalyst concentration was fixed at 25 μM. The concentrations of the peptides PrP(95–126) and PrP(95–114) were varied in the range 0–100 μM.

To determine the catalytic constants, the catalyst concentration (free Cu^2+^ or 1:1 Cu-PrP) was maintained constant at 25 μM, whereas the substrate concentration was varied between 0 and 4 mM.

The kinetic behavior of the Cu/peptide complexes was further investigated by varying SDS (0–20 mM) and LUV (0–1.5 mM) concentrations under the same experimental conditions. All measurements were performed in duplicate.

**CD studies of secondary structure of peptides.** Far-UV CD spectra of PrP(95–126) (10 µM), in the absence and presence of copper(II) ions (0–9.5 µM), were recorded in 5 mM phosphate buffer at pH 7.4 using a 1 cm path-length cell. Increasing concentrations of SDS (0–20 mM) were subsequently added to the preformed copper complex. The reverse order of addition was also examined by first evaluating the effect of SDS on the peptide alone in the buffer, followed by the addition of copper(II) to the solution. PrP structuring in aqueous solution was further investigated upon addition of increasing equivalents of copper (0–2.5 equiv.). Both PrP(95–126) alone and the Cu/PrP(95–126) complex were also analyzed in the presence of LUVs (100 µM, corresponding to the maximum concentration that avoided significant light scattering). CD spectra were recorded at a scanning rate of 100 nm/min with four accumulations.

**HPLC-ESI/MS studies.** The competitive peptide modification was studied by HPLC-ESI/MS, performing experiments in the same conditions used for kinetic studies. Samples were prepared by adding Cu^2+^ (25 μM), PrP(95–126) (25 μM) and MC (3 mM) in 50 mM HEPES buffer pH 7.4. Samples in membrane mimics were prepared by adding the same reagents but with further addition of SDS (20 mM) or LUVs (1.5 mM). Before LC-MS/MS analysis, SDS was precipitated by the addition of excess KCl: sulfuric acid was added to quench the reaction (to pH~2) at different reaction times, and, after standing for 30 min in ice, samples were centrifuged. The procedure was repeated twice, and then the soluble fraction was injected into LC-MS. The extraction of LUVs was performed with chloroform/butanol (1:1, *v*/*v*), allowing efficient partitioning of the lipids into the organic phase. Phospholipid detection was carried out in the organic phase by (i) acidic digestion to convert organic phosphorus into inorganic phosphate and (ii) total phosphorus determination. Briefly, the acidic digestion of organic phosphorus was performed by adding 70% perchloric acid, incubating the mixture at 200 °C for at least 30 min and adding hydrogen peroxide as clearing agent [74]. Total phosphorus determination was done by adding 10% ascorbic acid solution and 2.5% ammonium molybdate(VI) tetrahydrate to the digested samples and incubating at 100 °C for 10 min [72]. The results indicated that LUVs were efficiently extracted into the organic phase.

## 4. Conclusions

In this study, we expanded on our previous work on Cu/peptide systems, focusing on the redox activity of copper bound to prion protein fragments and their interactions with biologically relevant substrates such as catechols. The redox behavior of the Cu/PrP(95–126) complex shows only modest catalytic activation, comparable to the shorter PrP(106–114) fragment, which lacks His96. Binding affinity data support this observation and align with the literature suggesting that His96 is not involved in high-affinity copper binding [43,75].

Although the poor solubility of the full-length PrP above pH 6 represents a limitation for binding analysis [38,76], the logK values reported for Cu/PrP protein interaction are typically estimated in the range of 9–15, significantly higher than those obtained in the present work with PrP(95–126) [77,78,79].

Additionally, copper coordination to PrP peptides is strongly influenced by environmental factors, particularly membrane interactions. Electrostatic and hydrophobic interactions between peptides and lipid bilayers can modulate copper binding and reactivity. In membrane-mimicking systems (SDS micelles and lipid vesicles), we observed reduced oxidative reactivity toward external substrates such as catecholamines. This likely reflects a decrease in overall ROS production, alongside enhanced localized oxidative processes at key residues, especially His111 and Met109. The extent of these effects depends on both the lipid environment and the substrate, which may also interact with membranes.

Overall, the membrane environment exerts a dual effect: it protects surrounding biomolecules from oxidative damage while promoting localized oxidation within the PrP sequence. These findings highlight the critical role of the membrane context in regulating Cu/PrP redox chemistry and oxidative stress pathways.

This simplified in vitro system underscores the complexity of the molecular mechanisms involved in oxidative stress and may help explain discrepancies reported in the literature.

Further studies are needed to clarify how biological partners influence PrP metal binding, redox properties and PrP folding. These factors modulate the amyloidogenicity of the protein, not only through direct effects on ROS production but also indirectly by affecting processes such as protein cleavage and clearance [80,81,82].

## Figures and Tables

**Table 2:** Kinetic constants for the MC oxidation promoted by Cu/PrP complexes in HEPES buffer at pH 7.4 and 25 °C. * Values obtained by fitting data taken from Bacchella et al. [44].

	K_M_ (mM)	*k_cat_* (s^−1^)	*k_cat_*/K_M_ (M^−1^s^−1^)	Conditions
Cu^2+^	0.25 ± 0.04	(2.48 ± 0.02) × 10^−3^	9.92	In only HEPES buffer
[Cu^2+^-PrP(95–126)]	0.33 ± 0.03	(3.72 ± 0.04) × 10^−3^	11.27	
[Cu^2+^-PrP(106–114)] *	0.33 ± 0.04	(4.46 ± 0.1) × 10^−3^	13.51	
[Cu^2+^-PrP(84–114)] *	0.33 ± 0.07	(5.00 ± 0.2) × 10^−3^	15.15	
[Cu^2+^-PrP(76–114)] *	0.38 ± 0.04	(6.48 ± 0.2) × 10^−3^	17.05	
Cu^2+^	0.22 ± 0.02	(2.27 ± 0.05) × 10^−3^	10.32	In micellar SDS
[Cu^2+^-PrP(95–126)]	0.31 ± 0.03	(1.65 ± 0.07) × 10^−3^	5.32	

## Data Availability

The original contributions presented in this study are included in the article/Supplementary Material. Further inquiries can be directed to the corresponding author.

## Supplementary Materials

The following supporting information can be downloaded at: https://www.mdpi.com/article/10.3390/ijms27104184/s1.
