# Supplementary material for: Copper Coordination to the Prion Fragment (95–126): Implications for Neurodegenerative Diseases
Source: Int J Mol Sci. 2026 May 8;27(10):4184. doi: 10.3390/ijms27104184 (PMC13206253; doi:10.3390/ijms27104184)
Supplement: Supplementary file 1 [file ijms-27-04184-s001.zip › ijms-4229196-supplementary.pdf]

Supporting information

Copper coordination to the prion peptide PrP(95-126): Implications for neurodegenerative diseases.

Chiara Bacchella[1*], Angelo Ferraresi[1], Enrico Monzani[1], Simone Dell'Acqua[1*]

[1]Dipartimento di Chimica, Università di Pavia, Via Taramelli 12, 27100 Pavia, Italy
*Correspondence: chiara.bacchella@unipv.it (CB) simone.dellacqua@unipv.it (SD)
ORCID: 0000-0003-3256-8699 (CB); 0009-0006-5651-2410 (AF), 0000-0002-8791-6446 (EM); 0000-0002-1231-4045 (SD)

Material and Methods.

General. All chemicals used were of reagent grade and were obtained from Sigma-Aldrich, except for the lipids; 1-palmitoyl-2-oleoyl-sn-glycero-3-phosphocholine (POPC) and 1-palmitoyl-2-oleoyl-sn-glycero-3-phospho-L-serine (POPS) were purchased from Avanti Polar Lipids (Alabaster, AL, USA) and used as received. Preparative HPLC was performed using a Shimadzu LC-20AD Prominence instrument equipped with a diode array detector. Mass spectrometry and HPLC-MS/MS data were acquired with an LCQ ADV MAX ion-trap mass spectrometer, equipped with an ESI ion source. The instrument operated in automated LC-MS/MS mode, connected to a Surveyor HPLC system (Thermo Finnigan, San Jose, CA, USA) with a Phenomenex Jupiter 4u Proteo column (4 μm, 150×2.0 mm). Peptide fragment analysis was done using Bioworks 3.1 and Xcalibur 2.0.7 SP1 software (Thermo Finnigan, San Jose, CA, USA). UV-Vis titration and kinetic data were recorded on an Agilent 8453 diode array spectrophotometer equipped with a thermostated, magnetically stirred optical cell. Circular dichroism (CD) spectra were obtained in quartz cell of 1 cm path length by using a Jasco J-1500 spectropolarimeter.

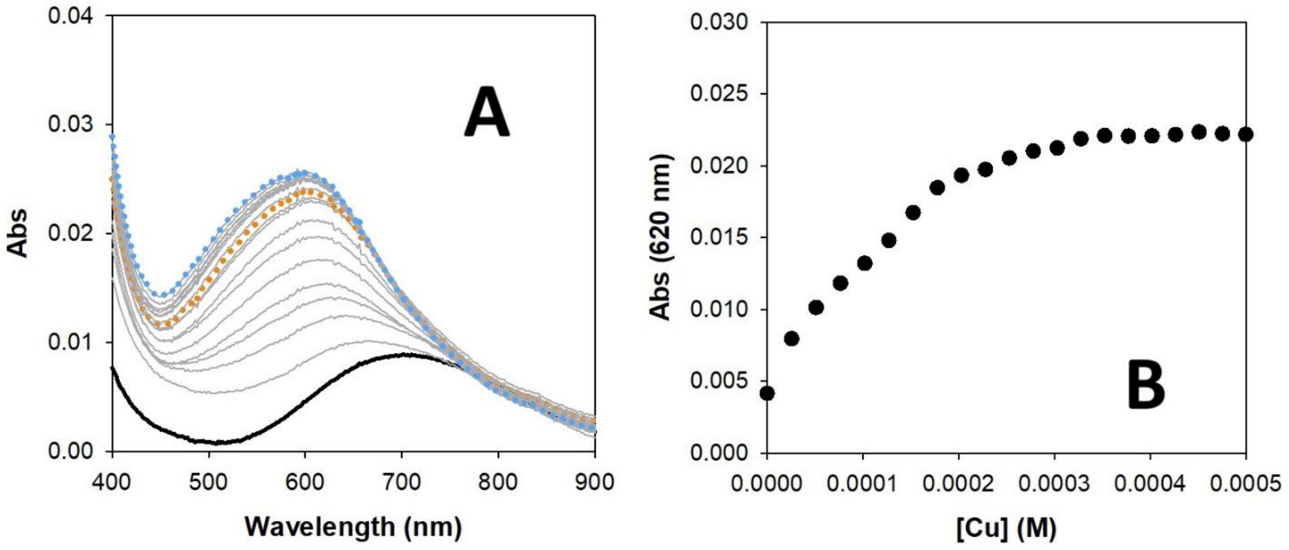

Figure S1. Titration of copper(II) solution (0.25 mM, black spectrum) with increasing equiv. of PrP(95-126) (0-1 mM) in 50 mM HEPES buffer at pH 7.4; dotted orange and ligth blue spectra show 1:1 and 1:2 Cu:PrP ratios, respectively. Panel B shows the experimental points extracted at 620 nm *vs* [PrP(95-126)].

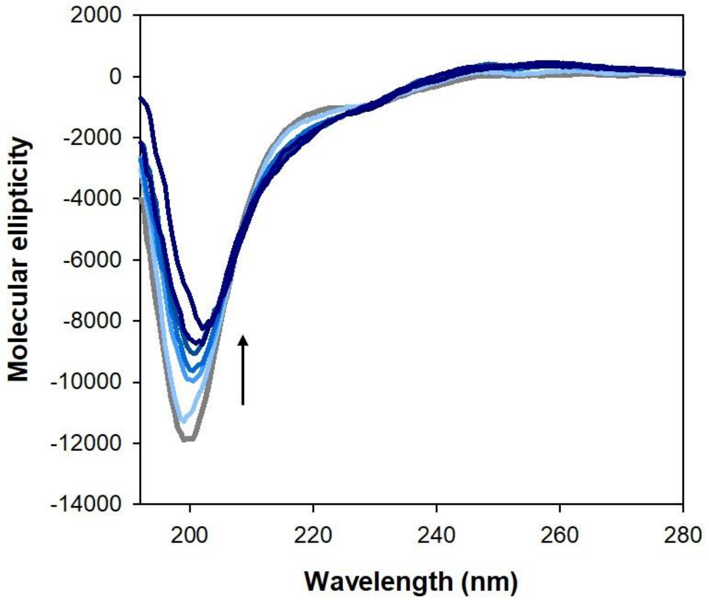

Figure S2. CD spectra in the far-UV region of PrP(95-126) alone (10 μM, grey spectrum) and adding increasing concentration of copper(II) (0-25 μM, scale of blue) in 5 mM phosphate buffer solution at pH 7.4.

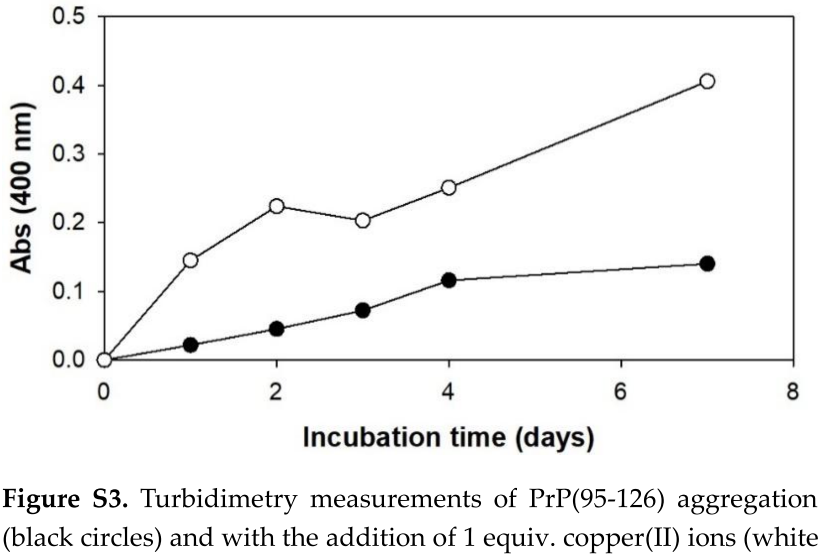

Figure S3. Turbidimetry measurements of PrP(95-126) aggregation monitored at 400 nm in PBS without (black circles) and with the addition of 1 equiv. copper(II) ions (white circles).

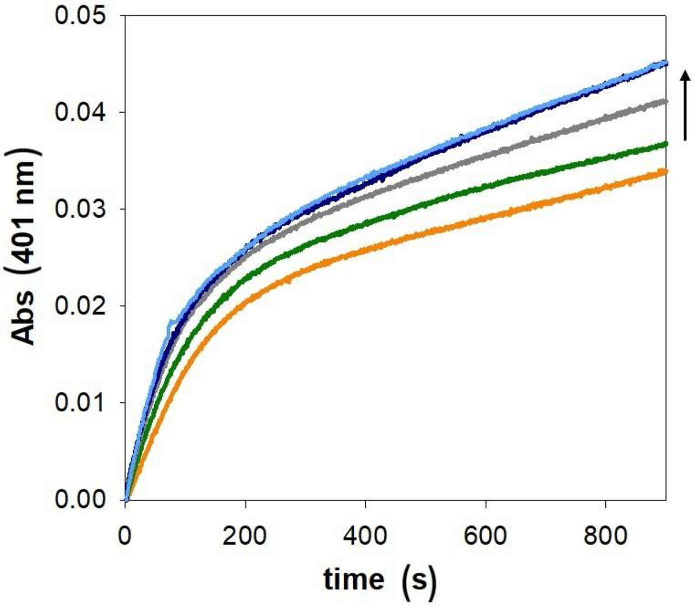

Figure S4. Absorbance changes with time in the MC (3 mM) oxidation in 50 mM HEPES buffer at pH 7.4 and 25 °C promoted by copper(II) alone (25 μM, orange trace) in the presence of PrP(95-114) (25 μM, green; 50 μM, grey; 75 μM, blue and 100 μM, light blue).

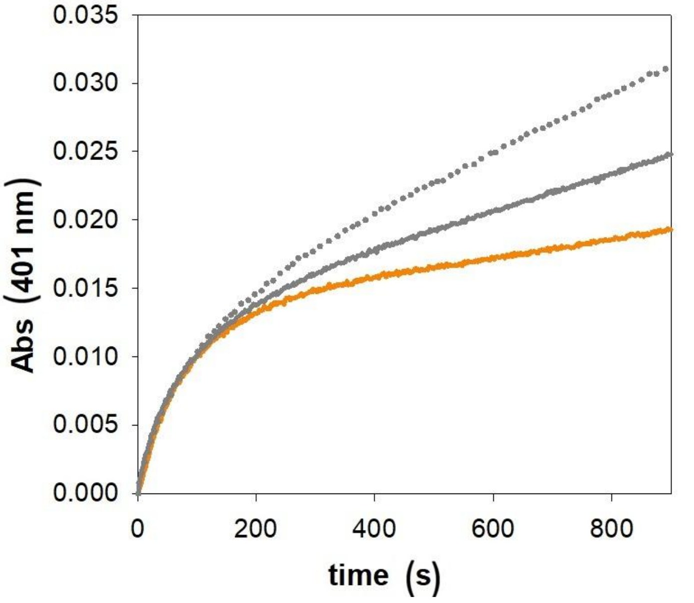

Figure S5. Absorbance changes with time in the MC (0.3 mM) oxidation in 50 mM HEPES buffer at pH 7.4 and 25 °C promoted by copper(II) alone (25 μM, orange trace) in the presence of PrP(95-126) (25 μM, solid grey and 50 μM, dotted grey).

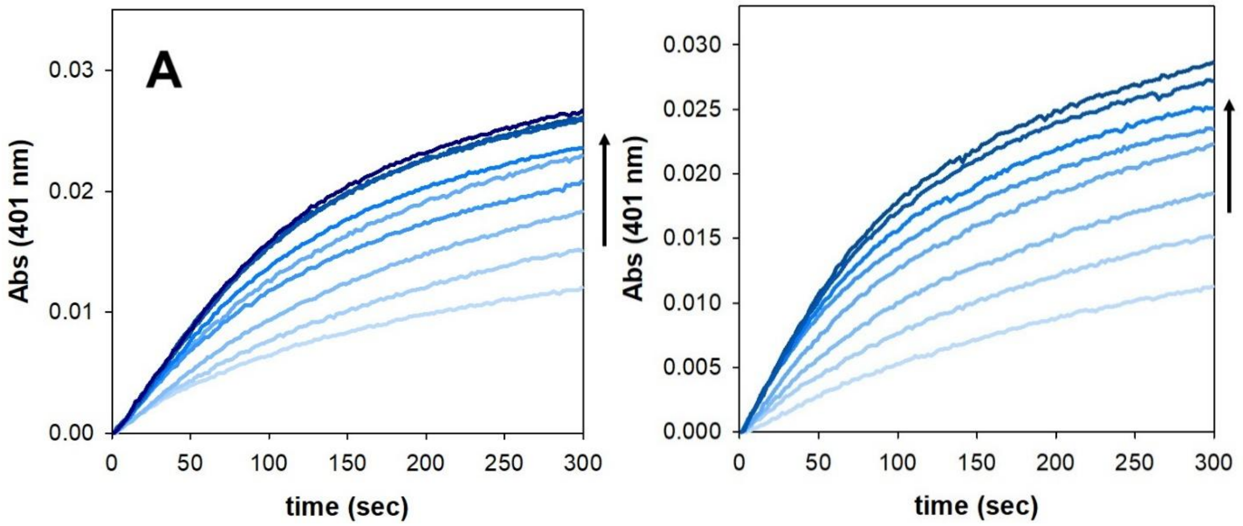

Figure S6. Absorbance changes with time in the MC oxidation in 50 mM HEPES buffer at pH 7.4 and 25 °C promoted by copper(II) alone (25 μM, panel A) or 1:1 [Cu-PrP(95-126)] complex (25 μM, panel B) by varying the substrate concentration in the range 0-4 mM.

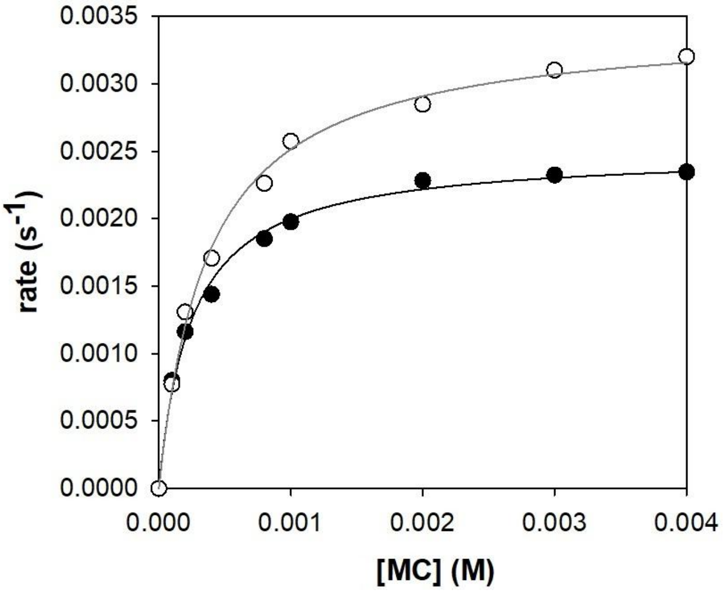

Figure S7. Initial oxidation catalyst turnover rates for MC oxidation in 50 mM HEPES buffer at pH 7.4 and 25 °C obtained in the presence of variable amounts of substrate (0-4 mM) and with copper alone (25 μM, black circles) and Cu-PrP(95-126) complex (1:1, 25 μM - white).

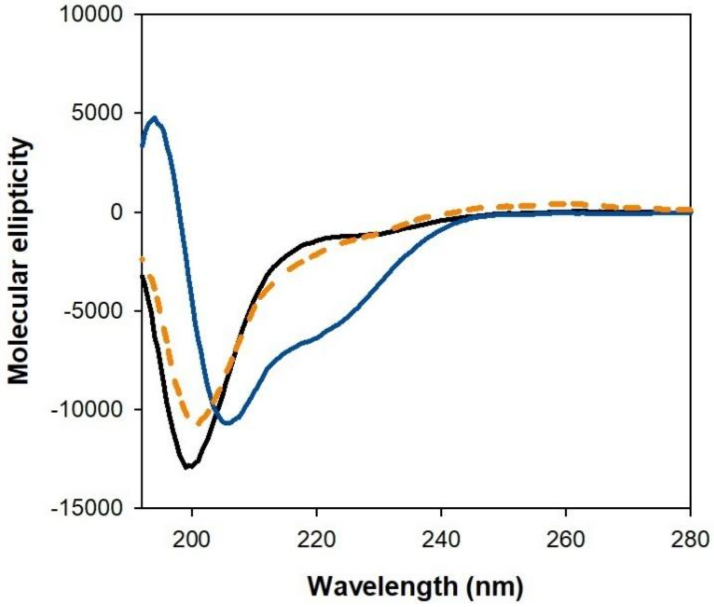

Figure S8. CD spectra in the far-UV region of PrP(95-126) alone (10 µM, black spectrum), after the addition of copper(II) (9.5 µM, dashed orange) and SDS (20 mM, blue) in 5 mM phosphate buffer solution at pH 7.4.

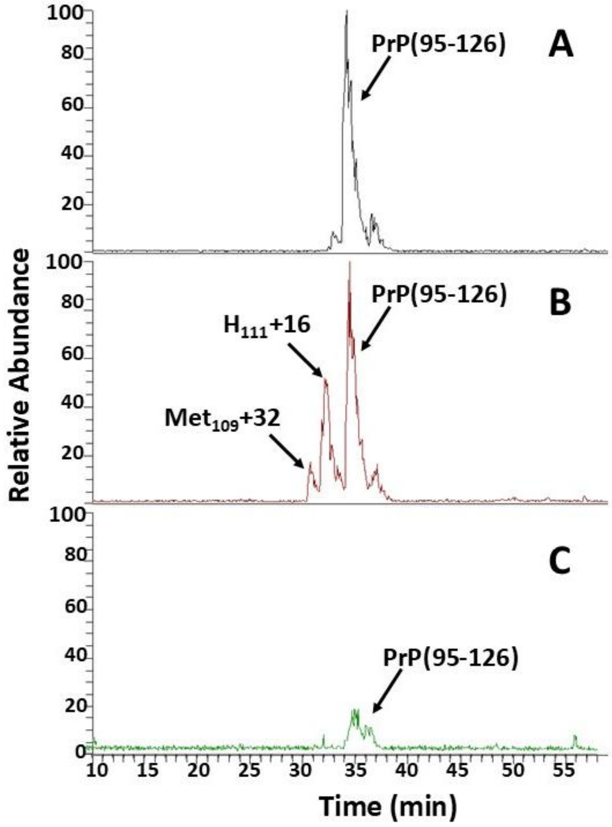

Figure S9. HPLC/MS analysis of oxidative modification of PrP(95-126) (25 µM) in the presence of **(A)** copper(II) (25 µM) and MC (3 mM), **(B)** in SDS micelle (20 mM) and **(C)** in LUVs (1.5 mM), in 10 mM HEPES buffer at pH 7.4. Modification after LUVs extraction was not determined because PrP(95-126) co-precipitated with phospholipids (~ 5% remained in solution).

Table S1. Modification with time of PrP(95-126) peptide (25 μM) detected by HPLC/MS analysis upon reaction with MC (3 mM) in the presence of Cu^{2+} (25 μM), SDS (20 mM) or LUVs (1.5 mM) in 10 mM HEPES buffer pH 7.4 at 20 °C.

Conditions	**Incubation time**	**PrP(95-126) (not modified)**	**PrP(95-126) mono-oxidized** (+16 on H_{111})	**PrP(95-126) bi-oxidized** (+32 on M_{109})	**PrP(95-126) covalently modified with Cat/Q** (+120, +122)
**Only buffer**	30′	84%	5%	1%	10%
	60′	78%	4%	2%	16%
**in SDS micelle**	30′	81%	10%	3%	6%
	60′	57%	29%	8%	6%
**in LUVs**		nd	nd	nd	nd

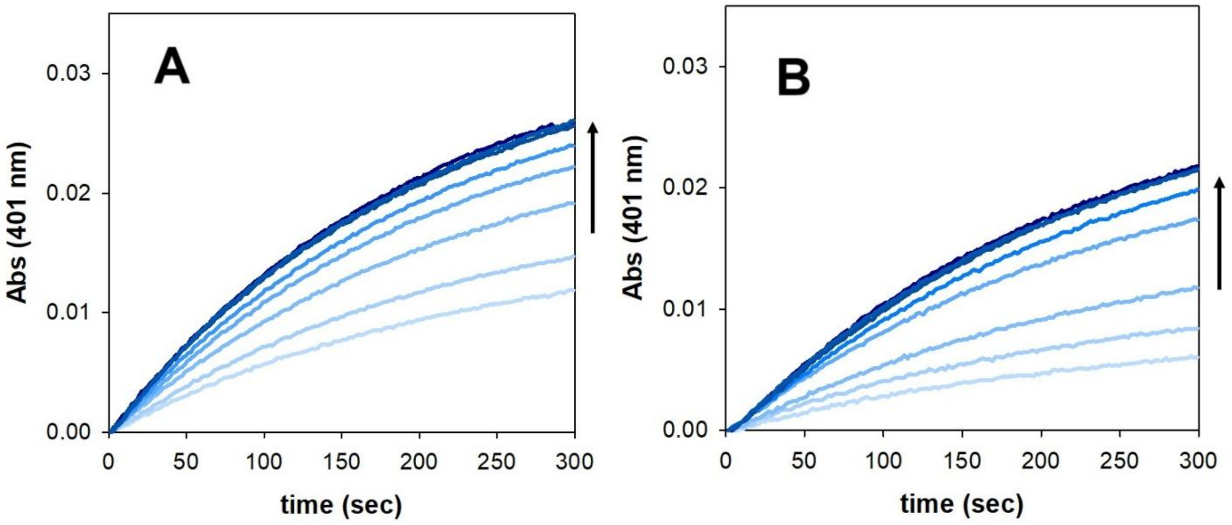

Figure S10. Absorbance changes with time in the MC (3 mM) oxidation in micellar solution of SDS (20 mM) in 50 mM HEPES buffer at pH 7.4 and 25 °C promoted by copper(II) alone (25 μM, panel A) or 1:1 [Cu-PrP(95-126)] complex (25 μM, panel B) by varying substrate concentration in the range 0-4 mM.

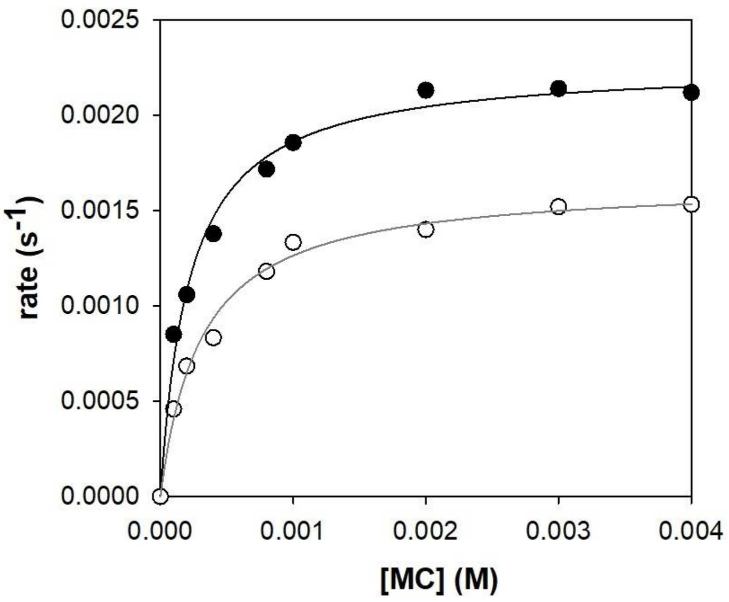

Figure S11. Initial oxidation catalyst turnover rates for MC oxidation in micellar solution of SDS (20 mM) in 50 mM HEPES buffer at pH 7.4 and 25 °C obtained in the presence of variable amounts of substrate (0-4 mM) and with copper alone (25 μM, black circles) or [Cu-PrP(95-126)] (1:1, 25 μM, white).

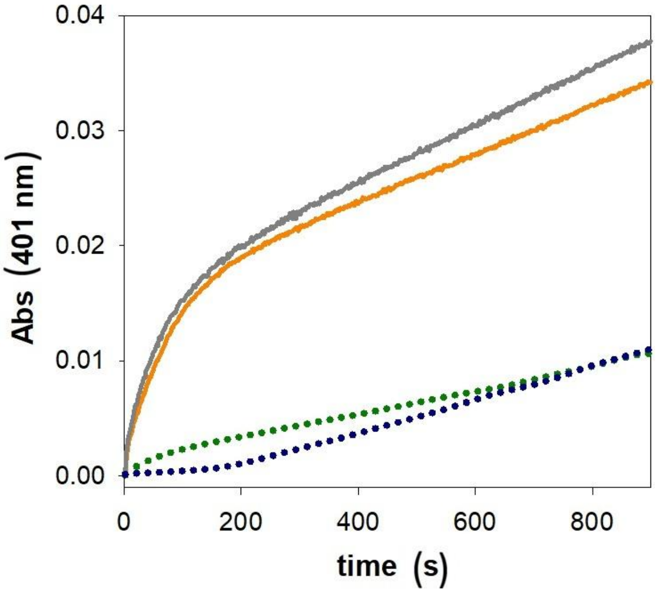

Figure S12. Absorbance changes with time in the MC (3 mM) oxidation in 50 mM HEPES buffer at pH 7.4 and 25 °C promoted by copper(II) alone (25 μM) without (orange trace) / with LUVs (1.5 mM, dotted green) and in the presence of PrP(95-126) (25 μM), without (grey) / with LUVs (1.5 mM, dotted blue).

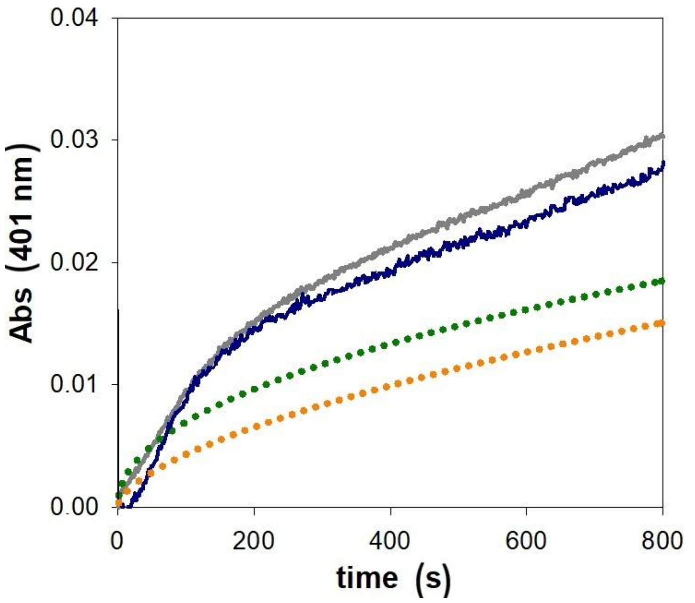

Figure S13. Absorbance changes with time in the MC (3 mM) oxidation in 50 mM HEPES buffer at pH 7.4 and 25 °C promoted by copper(I) alone (25 μM) without (grey trace) / with LUVs (1.5 mM, dotted green) and in the presence of PrP(95-126) (25 μM), without (blue) / with LUVs (1.5 mM, dotted orange).

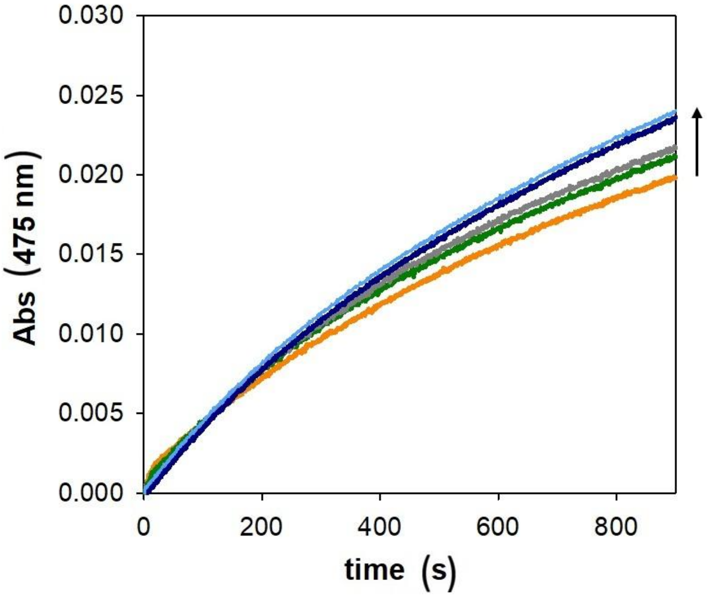

Figure S14. Absorbance changes with time in the dopamine (3 mM) oxidation in 50 mM HEPES buffer at pH 7.4 and 25 °C promoted by copper(II) alone (25 μM, orange trace) in the presence of PrP(95-126) (25 μM, green; 50 μM, grey; 75 μM, blue and 100 μM, light blue).
